# Supplementary material for: The replicative helicase CMG is required for the divergence of cell fates during asymmetric cell division in vivo
Source: Nat Commun. 2024 Oct 30;15:9399. doi: 10.1038/s41467-024-53715-2 (PMC11525967; doi:10.1038/s41467-024-53715-2)
Supplement: Supplementary file 1 — Supplementary Information [file 41467_2024_53715_MOESM1_ESM.pdf]

Supplementary Information

Fig. S1

A

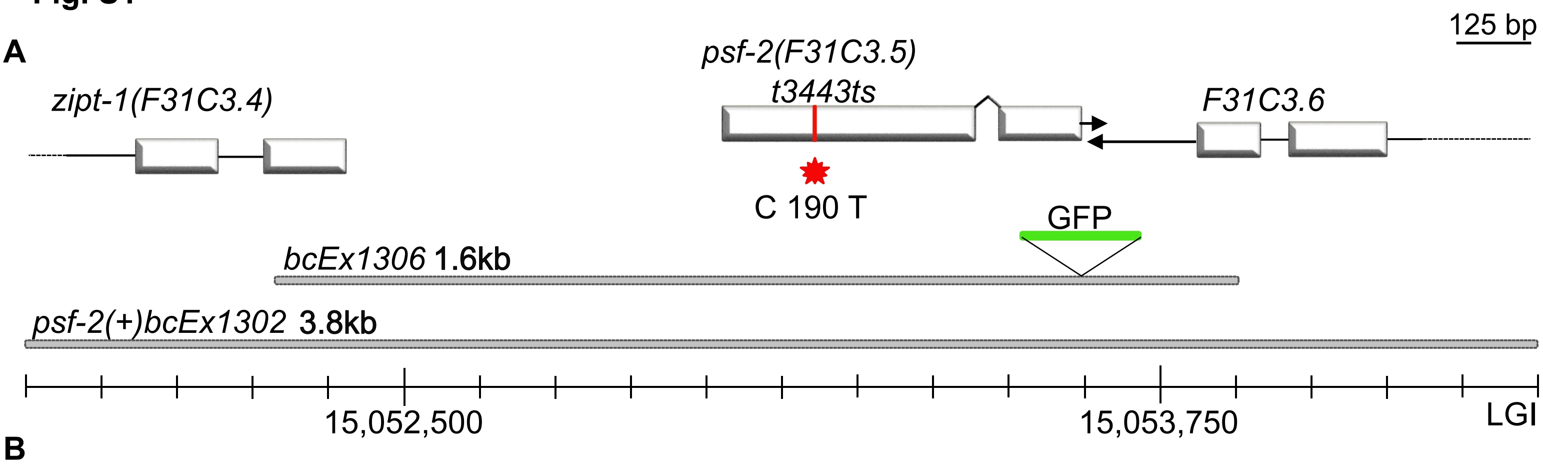

B

```
Sc      MSLPAHLQQTFSPEEIQFIVENEPIKIFPRITTRQKIRGDDRGTGNHTRWQLITTDDKAL    60
Ce      ----------MNAERCEFIAGNSLIEVIPSISDDRPIHL-------------ISGD---I    34
Dm      ----------MDPSIIEFIGEKCMISIIPNFSN-EPLHL-------------IYGP---V    33
Mm      ----------MDAAEVEFLAEKELVTIIPNFSL-DKIYL-------------IGGD---L    33
Hs      ----------MDAAEVEFLAEKELVTIIPNFSL-DKIYL-------------IGGD---L    33
                  :.    :*:  :  : ::* ::    :               *      :

                                     P 64 S
Sc      NNMVAMRSTEVVLWIALLLKQQSKCSIVAPQWLTTKELDRKIQYEKTHPDRFSELP-WNW    119
Ce      GPFEAGVPCRIPVWTAILMKRKHNCKVVAPQWMDVDELKKI-LTSETESQGLAKLP-DHF    92
Dm      GPFRAGFPVFVPLWMATHLRKQQKCRIVPPEWMDMDILEEI-KEEEKRSKFFTKMPCEHY    92
Mm      GPFNPGLPVDVPLWLAINLKQRQKCRLLPPEWMDVEKLEQM-RDEERKEETFTPVPSPHY    92
Hs      GPFNPGLPVEVPLWLAINLKQRQKCRLLPPEWMDVEKLEKM-RDHERKEETFTPMPSPYY    92
        . :       : :* *  :::: :* :: *:*:  . *..     : . . :: :*   :

Sc      LVLARILFNKAKDDFHDPIHELRGKIQDLREIRQIKVLKG----LKYLNESHLQLDNLSL    175
Ce      FEISHMLVRDAREDIFE-VEAVKSLVQDIYDRRDAKLRSSAIEFLRQNQTCHAQLDNVQL    151
Dm      MVVAQLVMSTAPDDVPR-CEELRTVIKDIFDIRESKLRTSIDAFIKGE-GTYAKLDNLTL    150
Mm      MEITKLLLNHASDNIPK-ADTIRTLIKDLWDTRMAKLRVSADSFVRQQ-EAHAKLDNLTL    150
Hs      MELTKLLLNHASDNIPK-ADEIRTLVKDMWDTRIAKLRVSADSFVRQQ-EAHAKLDNLTL    150
        : :::::.  * ::.    . ::  ::*: : *  *:  .    ::     : :***: *

Sc      LEINELRPFITEIMDKLREIHTASLTAGTENDE------------E---EFNI           213
Ce      IEASSARATLEACRQMGAVV-RNKHESTPL-----------------------           180
Dm      LEIHSVRPILPYSLDHIARYQRTATASQRDTSMLSASMAGSSSGPNSNSLFSQ           203
Mm      MEISSSGAFLTQALNHMYKLRTNLQPSESTQSQ---DF---------------           185
Hs      MEINTSGTFLTQALNHMYKLRTNLQPLESTQSQ---DF---------------           185
        :*       :    :
```

C

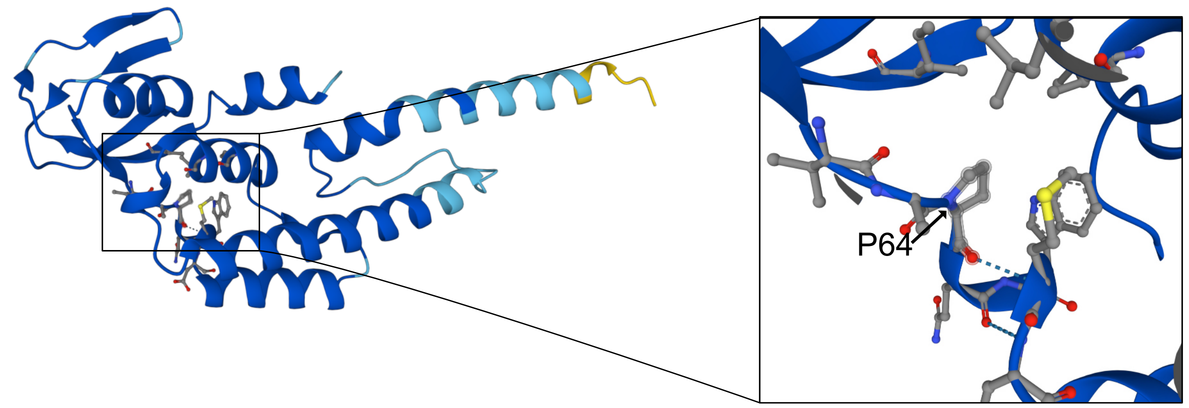

Supplementary Figure 1. Identification of *t3443*ts mutation in *psf-2* locus and alignment of PSF-2 protein sequence with orthologs in different species. (A) Top. Schematic of *psf-2* locus (based on (www.wormbase.org[1,2]) and adjacent transcription units on LGI. *t3443*ts is a C-to-T change at position 190bp of *psf-2*'s coding sequence and is indicated in red. Bottom. Schematic of 3.8kb and 1.6kb genomic fragments that were used to generate the rescuing transgenes *bcEx1302* and *bcEx1306*. **(B)** Alignment of PSF-2 protein sequence with orthologs of PSF-2 in different species generated using Clustal Omega[3]. *t3443*ts causes a predicted proline-to-serine change at position 64 of PSF-2's amino acid sequence and is indicated in red. **(C)** Structure of *C. elegans* PSF-2 generated with AlphaFold[4,5]. An enlargement of the region around Proline 64 (P64) is shown on the right.

Fig. S2

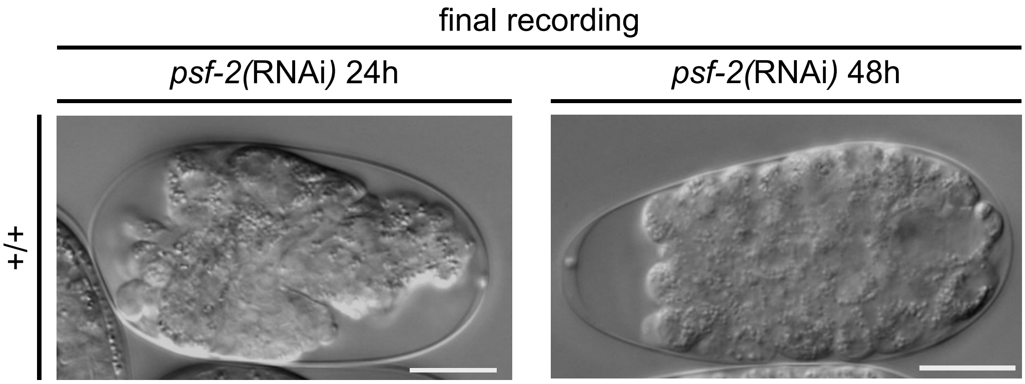

Supplementary Figure 2. The severity of the *psf-2*(RNAi) phenotype is dependent on the duration of RNAi knock-down. DIC images of the final recordings for representative embryos. After a 24h RNAi treatment (left), embryos reach and initiate the morphogenesis stage. After a 48h RNAi treatment (right), embryos are unable to reach and initiate the morphogenesis stage. Instead, they arrest after reaching the ~50-cell stage. Scale bars 10 μM.

Fig. S3

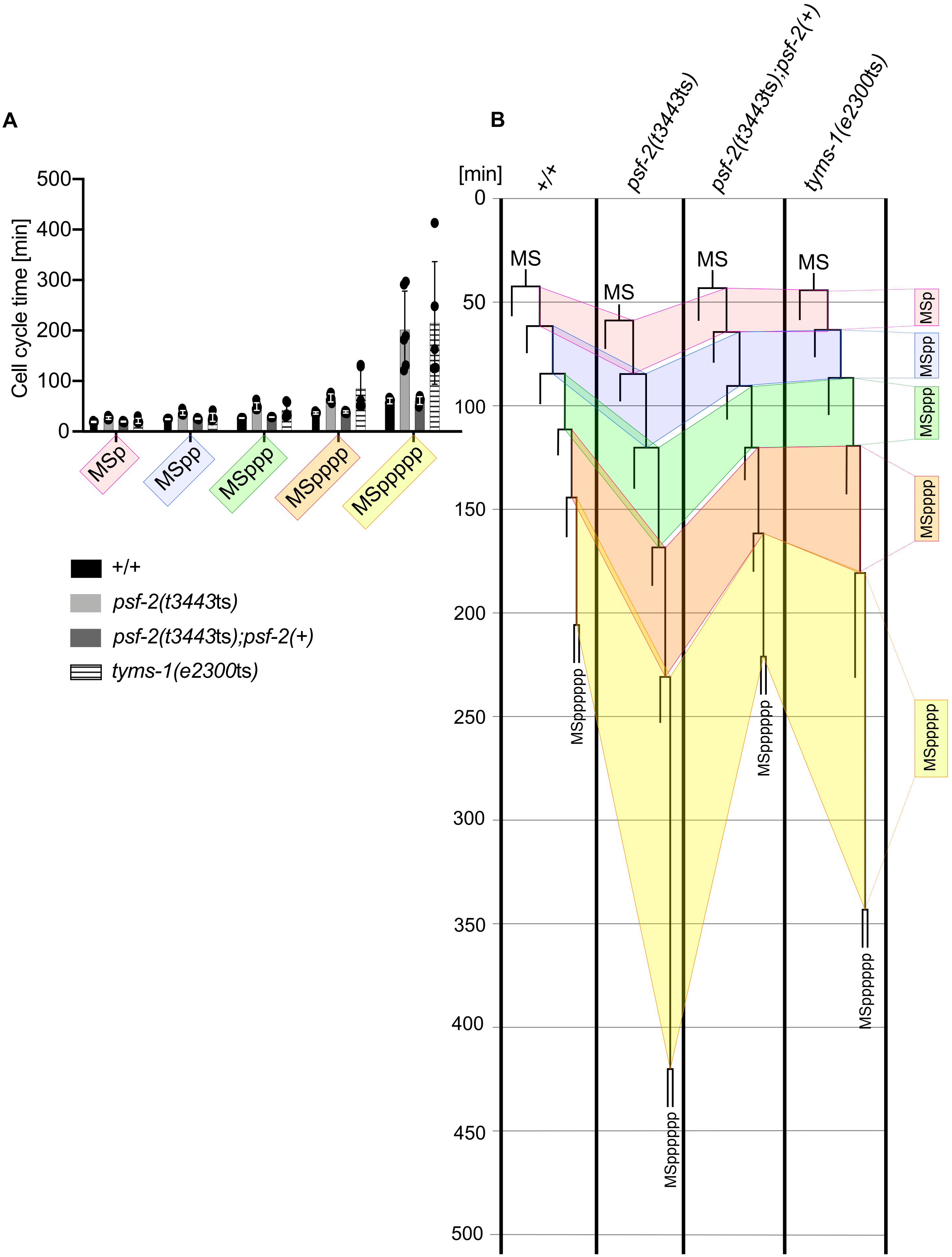

A
Cell cycle time [min]
500
400
300
200
100
0
MSp
MSpp
MSppp
MSpppp
MSppppp
+/+
psf-2(t3443ts)
psf-2(t3443ts);psf-2(+)
tyms-1(e2300ts)
B
[min]
0
50
100
150
200
250
300
350
400
450
500
+/+
psf-2(t3443ts)
psf-2(t3443ts);psf-2(+)
tyms-1(e2300ts)
MS
MS
MS
MS
MSp
MSpp
MSppp
MSpppp
MSppppp
MSpppppp
MSpppppp
MSpppppp
MSpppppp

Supplementary Figure 3. The increase in cell cycle length in *psf-2(t3443*ts) is not lineage-dependent. (A) Cell cycle length [min] of the MSppppp cell and its ancestors in wild-type (+/+) (n=6), *psf-2*(*t3443*ts) (n=6), *psf-2*(*t3443*ts); *psf-2*(+) (transgene *bcEx1302*) (n=5) and *tyms-1*(*e2300*ts) (n=5) animals at 25°C. Mean ±SD are indicated. **(B)** MSpppppp lineage of representative animals of the genotypes indicated. The wild-type and *psf-2*(*t3443*ts); *psf-2*(+) embryos analyzed completed embryogenesis and hatched. Analyses were performed at 25 °C and *tyms-1(e2300*ts*)* embryos were shifted to 25 °C at the 1-4-cell stage.

Fig. S4

A

Cell	+/+	*psf-2(t3443*ts) #1	#2	#3	#4
ABalaapapp	cell division	cell division	cell division	cell division	n. a.
ABalaappap	cell division	cell division blocked	cell division	cell division	n. a.
ABalapapap	cell division	cell division	cell division blocked	cell division	cell division blocked
ABalappaap	cell division	cell division blocked	cell division	cell division	n. a.
ABalppaaap	cell division	cell division	cell division	cell division	cell division
ABalppaapp	cell division	cell division blocked	cell division	cell division	cell division blocked
ABaraaaapa	cell division	cell division	cell division blocked	cell division	?
ABarpaaapa	cell division	cell division blocked	cell division blocked	?	?
ABplpappaa	cell division	cell division	cell division	cell division	cell division blocked
ABplppaaaa	cell division	cell division	cell division blocked	?	?
ABplpppapa	cell division	cell division	cell division	cell division	cell division blocked
ABprppaaaa	cell division	cell division	?	?	?
ABprpppapa	cell division	cell division blocked	cell division	cell division blocked	cell division

cell division; cell division blocked
? cell lost; n.a. not applicable

B

Genotype	% cell division	% cell death	n
+/+	100	0	51
*psf-2(t3443*ts)	65.8	0	41

C

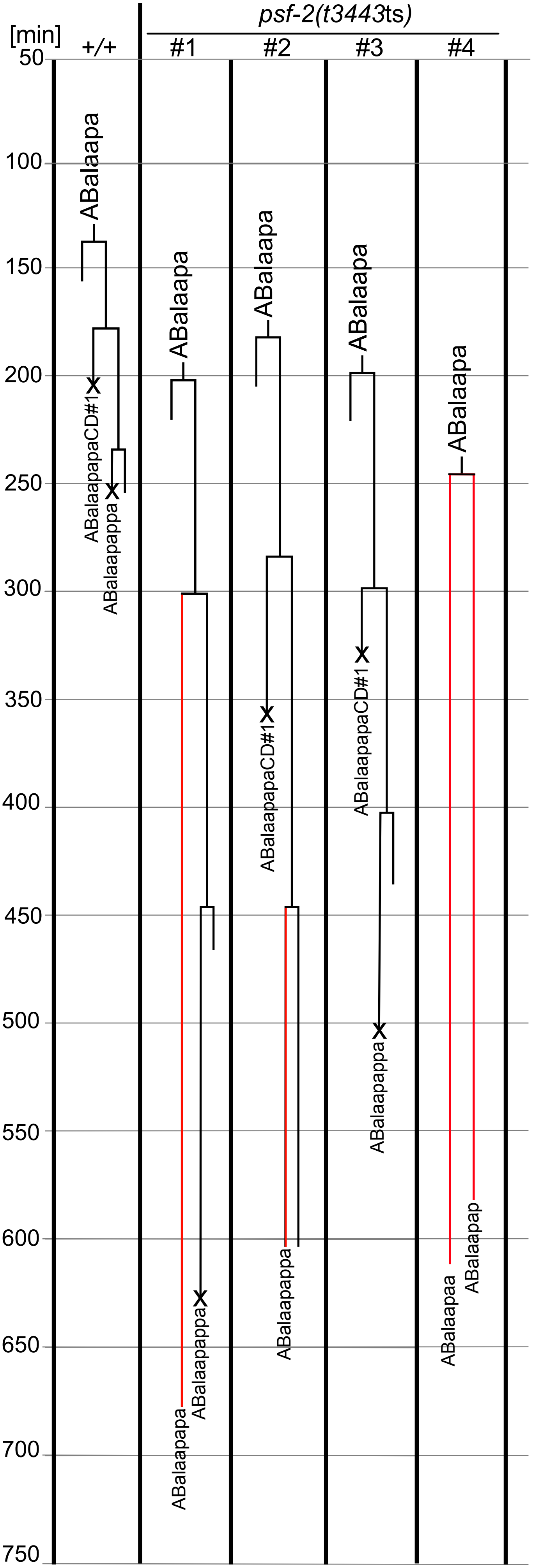

Supplementary Figure 4. Identification of the cell fates of the sisters of the 13 AB-derived 1st wave cell deaths. **(A)** Detailed cell fate analysis of the sister cells shown in Figure 2A in wild-type (+/+) (n=4; data from one representative embryo is shown), *psf-2(t3443*ts*)* (n=4). Cell fate was determined based on 4D lineaging analyses performed on long-term recordings done at 25°C as described in Methods. **(B)** Percentage [%] cell division of sisters of the first wave of cell death (13 AB-derived cell deaths). Summary of data shown in **(A)**. **(C)** Lineage of the ABalaapa cell and its descendants in wild-type (+/+) and in the four *psf-2*(*t3443*ts) analyzed embryos shown in **(A)** and summarized **(B)** and **Fig. 2A**. Red lineages indicate blocked cell divisions

Fig. S5

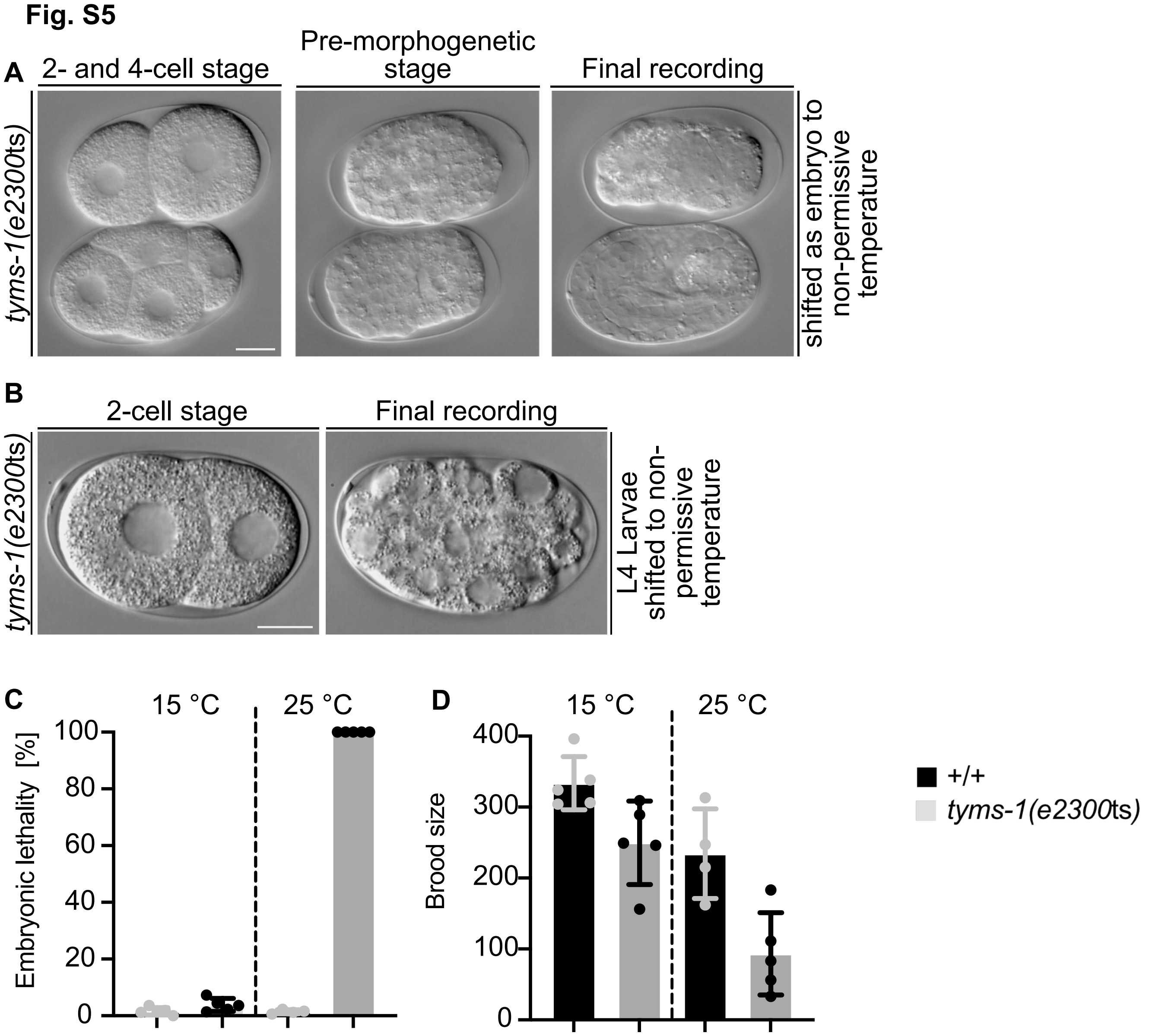

E

```
Sc --------------------------MTMDGKNKEEEQYLDLCKRIIDEGEFRPDRTGTGTLSLFAPPQLRFSLRDDTFPLLTTKK 60
Ce ----MN---KENI-IADAPSDVVKTVQQQVHLNQDEYKYLKQVEQILREGTRRDDRTGTGTISIFG-MQSKYCLRNGTIPLLTTKR 77
Dm MVLTPTKDGPDQESMPLPADNGESPSKQQAPVNRDEMHYLDLLRHIIANGEQRMDRTEVGTLSVFG-SQMRFDMRN-SFPLLTTKR 84
Mm ----MLVVGSELQSDA------QQL--SAEAPRHGELQYLRQVEHILRCGFKKEDRTGTGTLSVFG-MQARYSLRD-EFPLLTTKR 72
Hs ----MPVAGSELPRRPLPPAAQERD--AEPRPPHGELQYLGQIQHILRCGVRKDDRTGTGTLSVFG-MQARYSLRD-EFPLLTTKR 78
                                                    : * :**    .:*:  *   : *** .**:*:*.  * :: :*:  :******:
```

W 80 C

```
Sc VFTRGIILELLWFLAGDTDANLLSEQGVKIWDGNGSREYLDKMGFKDRKVGDLGPVYGFQWRHFGAKYKTCDDDYTGQGIDQLKQV 146
Ce VYWKGVLEELLWFISGSTDGKLLMEKNVKIWEKNGDRAFLDNLGFTSREEGDLGPVYGFQWRHFGAKYVDCHTDYSGQGVDQLAEV 163
Dm VFFRAVAEELLWFVAGKTDAKLLQAKNVHIWDGNSSREFLDKMGFTGRAVGDLGPVYGFQWRHFGAQYGTCDDDYSGKGIDQLRQV 170
Mm VFWKGVLEELLWFIKGSTNAKELSSKGVRIWDANGSRDFLDSLGFSARQEGDLGPVYGFQWRHFGAEYKDMDSDYSGQGVDQLQKV 158
Hs VFWKGVLEELLWFIKGSTNAKELSSKGVKIWDANGSRDFLDSLGFSTREEGDLGPVYGFQWRHFGAEYRDMESDYSGQGVDQLQRV 154
   *: :.:  *****: *.*:.: *  :.*:**: *..* :**.:**. *  ****************:*    .**:*:*:*** .*

Sc IHKLKTNPYDRRIIMSAWNPADFDKMALPPCHIFSQFYVSFPKEGEGSGKPRLSCLLYQRSCDMGLGVPFNIASYALLTRMIAKVV 233
Ce IRQIKEQPDSRRIIMSAWNPSDLGQMVLPPCHTMCQFYVD---------NGELSCQLYQRSGDMGLGVPFNLASYGLLTHMIAKVC 241
Dm IDTIRNNPSDRRIIMSAWNPLDIPKMALPPCHCLAQFYVSEK-------RGELSCQLYQRSADMGLGVPFNIASYALLTHMIAHVT 250
Mm IDTIKTNPDDRRIIMCAWNPKDLPLMALPPCHALCQFYVV---------NGELSCQLYQRSGDMGLGVPFNIASYALLTYMIAHIT 236
Hs IDTIKTNPDDRRIIMCAWNPRDLPLMALPPCHALCQFYVV---------NSELSCQLYQRSGDMGLGVPFNIASYALLTYMIAHIT 232
   *  :: :* .*****.**** *:  *.*****

Sc DMEPGEFIHTLGDAHVYKDHIDALKEQITRNPRPFPKLKIKRDVKDIDDFKLTDFEIEDYNPHPRIQMKMSV 304
Ce GLKPGTLVHTLGDAHVYSNHVDALKIQLDREPYAFPKIRFTRDVASIDDFTSDMIALDDYKCHPKIPMDMAV 312
Dm GLKPGDFVHTMGDTHVYLNHVEPLKEQLERTPRPFPKLIIKRQVQDIEDFRFEDFQIVDYNPHPKIQMDMA- 320
Mm GLQPGDFVHTLGDAHIYLNHIEPLKIQLQREPRPFPKLKILRKVETIDDFKVEDFQIEGYNPHPTIKMEMAV 307
Hs GLKPGDFIHTLGDAHIYLNHIEPLKIQLQREPRPFPKLRILRKVEKIDDFKAEDFQIEGYNPHPTIKMEMAV 313
   .::** ::**:**:*:* :*:: ** *: * *  ***: : *.*  *:**    : : .*: ** * *.*:
```

Supplementary Figure 5. Identification of *e2300*ts mutation in *tyms-1* locus and alignment of TYMS-1 protein sequence with orthologs in different species. (A) DIC images of representative *tyms-1*(*e2300*ts) embryos at the 2- and 4-cell stage, the pre-morphogenetic stage and at the final recording (terminal phenotype). Embryos were shifted to 25°C at the 2- to 4-cell stage. **(B)** DIC images of representative *tyms-1*(*e2300*ts) embryos at the 2-cell stage and at the final recording (terminal phenotype). L4 larvae were shifted to 25°C, 2-cell stage embryos were extracted after 16h and mounted for long-term imaging. For both (A) and (B), images were taken from long-term recordings performed at 25°C. Scale bars represent 10 µM. **(C)** Embryonic lethality [%] and **(D)** Brood size at permissive (15°C) and non-permissive temperature (25°C) in wild-type (+/+) and *tyms-1*(*e2300*ts). Embryonic lethality and brood size of four (n=4) or five (n=5) adults were analyzed in the case of wild-type (15°C or 25°C, respectively). In the case of *tyms-1*(*e2300*ts), embryonic lethality and brood size of five adults (n=5) were analyzed. Mean ±SD are indicated. **(E)** Alignment of the TYMS-1 protein sequence with orthologs of thymidylate synthetase in different species generated using Clustal Omega[3].

Fig. S6

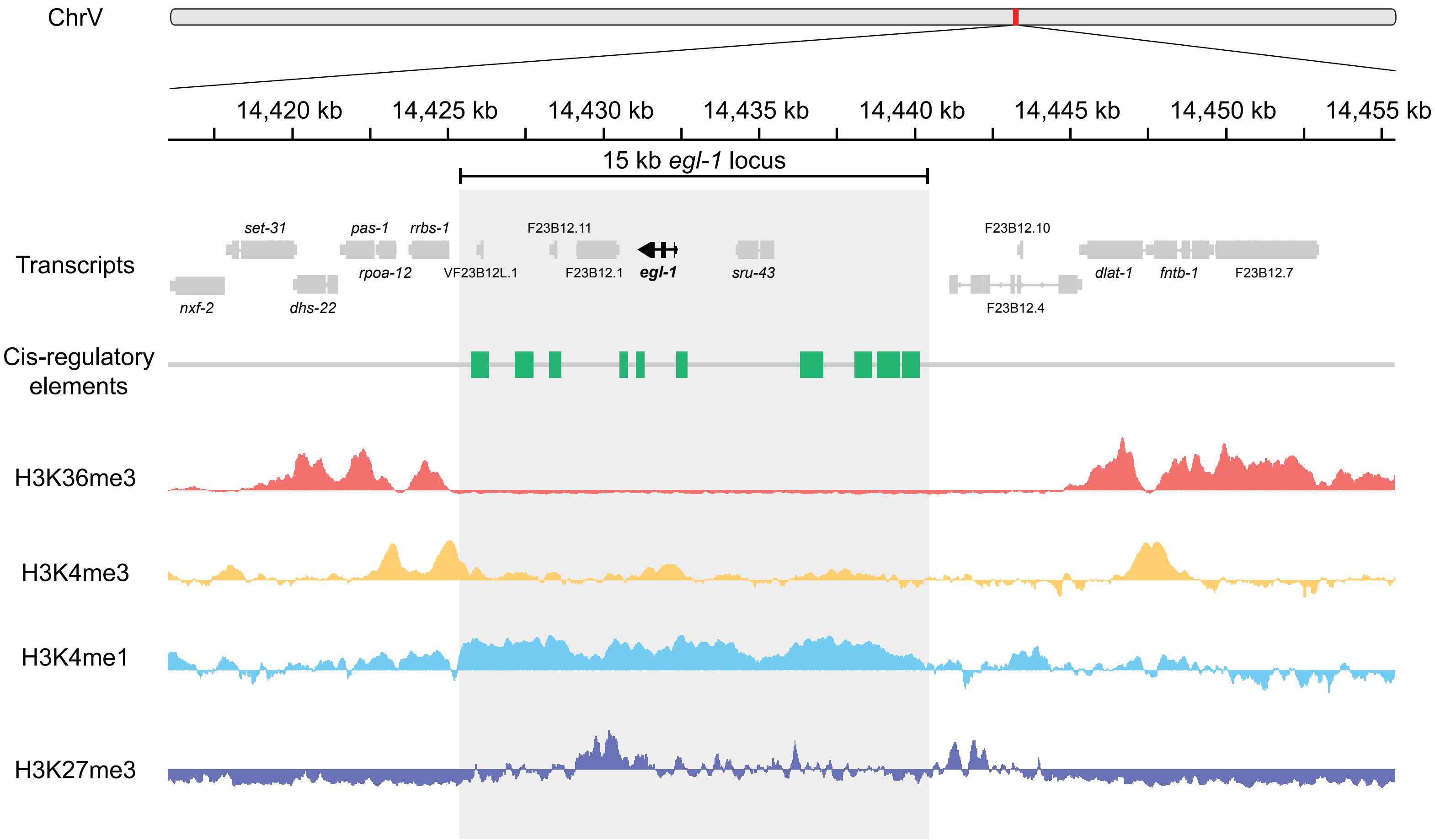

ChrV
14,420 kb
14,425 kb
14,430 kb
14,435 kb
14,440 kb
14,445 kb
14,450 kb
14,455 kb
15 kb egl-1 locus
Transcripts
set-31
pas-1
rrbs-1
F23B12.11
F23B12.10
rpoa-12
VF23B12L.1
F23B12.1
egl-1
sru-43
dlat-1
fntb-1
F23B12.7
nxf-2
dhs-22
F23B12.4
Cis-regulatory
elements
H3K36me3
H3K4me3
H3K4me1
H3K27me3

Supplementary Figure 6. Genome tracks of ChrV:14,415,000-14,455,000 (40 kb)

Within the 40 kb view, the 15 kb *egl-1* locus containing the transcript and cis-regulatory elements is highlighted in grey. All transcripts (www.wormbase.org)[1,2] within the 40 kb locus are indicated in grey, except the *egl-1* transcript, which is indicated in black. The *cis*-regulatory elements of the *egl-1* locus are indicated in green[6]. ChIP-seq peaks obtained from bulk *C. elegans* embryos for various histone H3 modifications were mined from publicly available datasets[7]. H3K36me3 tracks are indicated in red, H3K4me3 tracks are indicated in yellow, H3K4me1 tracks are indicated in light blue and H3K27me3 tracks are indicated in dark blue. This figure was adapted from the Integrative Genomics Viewer Application https://igv.org/app/[8] .

Supplementary Table S1 *C. elegans* strains used

Strain name	Genotype	Source
N2	+/+	CGC
GE746	*tyms-1(e2300)*	CGC as GE31
MD3979	*psf-2(t3443*ts*)*	this study
MD4114	*psf-2(t443*ts*); bcEx1302*	this study
MD4125	*psf-2(t3443*ts*); bcEx1306*	this study
MD3832	*bcIs133*	[9]
MD4865 or 4856	*psf-2(t3443*ts*) I; bcIs133*	this study
MT19454	*nIs396*	[10]
MD4875	*psf-2(t3443*ts*) I; nIs396*	this study
MD4876	*his-9(n5357) II; nIs396*	this study
CHL154	*saIs14[lin-48p::gfp] II; otIs356[rab-3prom::NLS::rfp], him-5(e1490)*	this study
CHL155	*psf-2(t3443*ts*) I; saIs14[lin-48p::gfp] II; otIs356[rab-3prom::NLS::rfp], him-5(e1490)*	this study
CHL156	*psf-2(t3443*ts*) I; saIs14[lin-48p::gfp] II; otIs356[rab-3prom::NLS::rfp], him-5(e1490) V; bcEx1306[pBC1695(psf-2(wt)::GFP)]*	this study

Reference

1. Davis, P. et al. WormBase in 2022-data, processes, and tools for analyzing *Caenorhabditis elegans*. Genetics 220 (2022). https://doi.org/10.1093/genetics/iyac003
2. Sternberg, P. W. et al. WormBase 2024: status and transitioning to Alliance infrastructure. Genetics (2024). https://doi.org/10.1093/genetics/iyae050
3. Madeira F., Madhusoodanan N., Lee J., Eusebi A., Niewielska A., Tivey A.R.N., Lopez R., Butcher S. The EMBL-EBI Job Dispatcher sequence analysis tools framework in 2024. *Nucleic Acids Research* **52**, 521-525 (2024). https://doi.org/10.1093/nar/gkae241
4. Jumper, J. et al. Highly accurate protein structure prediction with AlphaFold. *Nature* **596**, 583-589 (2021). https://doi.org/10.1038/s41586-021-03819-2
5. Varadi, M. & Velankar, S. The impact of AlphaFold Protein Structure Database on the fields of life sciences. *Proteomics* **23**, e2200128 (2023). https://doi.org/10.1002/pmic.202200128
6. Conradt, B., Wu, Y. C. & Xue, D. Programmed Cell Death During *Caenorhabditis elegans* Development. *Genetics* **203**, 1533-1562 (2016). https://doi.org/10.1534/genetics.115.186247
7. Janes, J. et al. Chromatin accessibility dynamics across *C. elegans* development and ageing. Elife 7 (2018). https://doi.org/ARTN e3734410.7554/eLife.37344
8. Robinson, J. T. et al. Integrative genomics viewer. *Nat Biotechnol* **29**, 24-26 (2011). https://doi.org/10.1038/nbt.1754
9. Mishra N., Wei H. and Conradt B. *Caenorhabditis elegans ced-3* Caspase Is Required for Asymmetric Divisions That Generate Cells Programmed To Die*. Genetics* ***210*** *(*2018 *(*PMID: 30194072*)*
10. Nakano S., Ellis R.E., Horvitz H. R. Otx-dependent expression of proneural bHLH genes establishes a neuronal bilateral asymmetry in C. elegans. *Development* **137**, 4017-4027 (2010) https://doi.org/10.1242/dev.058834 (PMID: 21041366)
